# Usability Assessment of the Missouri Cancer Registry’s Published Interactive Mapping Reports: Round One

**DOI:** 10.2196/humanfactors.7899

**Published:** 2017-08-04

**Authors:** Awatef Ahmed Ben Ramadan, Jeannette Jackson-Thompson, Chester Lee Schmaltz

**Affiliations:** ^1^ Department of Health Management and Informatics (HMI) University of Missouri-Columbia Columbia, MO United States; ^2^ University of Missouri Informatics Institute (MUII) University of Missouri-Columbia Columbia, MO United States; ^3^ Missouri Cancer Registry and Research Center (MCR-ARC) University of Missouri-Columbia Columbia, MO United States

**Keywords:** geographic information systems, health professionals, interactive maps, Missouri Cancer Registry, usability

## Abstract

**Background:**

Many users of spatial data have difficulty interpreting information in health-related spatial reports. The Missouri Cancer Registry and Research Center (MCR-ARC) has produced interactive reports for several years. These reports have never been tested for usability.

**Objective:**

The aims of this study were to: (1) conduct a multi-approach usability testing study to understand ease of use (user friendliness) and user satisfaction; and (2) evaluate the usability of MCR-ARC’s published InstantAtlas reports.

**Methods:**

An institutional review board (IRB) approved mixed methodology usability testing study using a convenience sample of health professionals. A recruiting email was sent to faculty in the Master of Public Health program and to faculty and staff in the Department of Health Management and Informatics at the University of Missouri-Columbia. The study included 7 participants. The test included a pretest questionnaire, a multi-task usability test, and the System Usability Scale (SUS). Also, the researchers collected participants’ comments about the tested maps immediately after every trial. Software was used to record the computer screen during the trial and the participants’ spoken comments. Several performance and usability metrics were measured to evaluate the usability of MCR-ARC’s published mapping reports.

**Results:**

Of the 10 assigned tasks, 6 reached a 100% completion success rate, and this outcome was relative to the complexity of the tasks. The simple tasks were handled more efficiently than the complicated tasks. The SUS score ranged between 20-100 points, with an average of 62.7 points and a median of 50.5 points. The tested maps’ effectiveness outcomes were better than the efficiency and satisfaction outcomes. There was a statistically significant relationship between the subjects’ performance on the study test and the users’ previous experience with geographic information system (GIS) tools (*P*=.03). There were no statistically significant relationships between users’ performance and satisfaction and their education level, work type, or previous experience in health care (*P*>.05). There were strong positive correlations between the three measured usability elements.

**Conclusions:**

The tested maps should undergo an extensive refining and updating to overcome all the discovered usability issues and meet the perspectives and needs of the tested maps’ potential users. The study results might convey the perspectives of academic health professionals toward GIS health data. We need to conduct a second-round usability study with public health practitioners and cancer professionals who use GIS tools on a routine basis. Usability testing should be conducted before and after releasing MCR-ARC’s maps in the future.

## Introduction

Geographic information system (GIS) tools should be planned to achieve the desires and perceptions of the tools’ targeted users. The development of GIS tools does not seem to be an issue; the problem seems to be their effective and efficient use [1,2].

Health care and public health fields have started using sophisticated technology to analyze and visualize health-related databases. Advanced visualization technology is becoming essential and important nationally and internationally to help control many health-related problems. This technology has positively impacted health-related research and policy development. Therefore, these databases need to be held wisely, investigated sufficiently to produce consistent results, not mislead the audiences, and produce the expected impact [3].

As the previous literature has pointed out, high percentages of any new digital technology’s potential users find difficulties in interpreting and understanding the associated complicated and combined information [4-6]. For the GIS tools where statistical and spatial information are combined, users have faced similar difficulties. Several reasons have been identified: inadequate experience and training on how to use the technology; lack of awareness among potential users; refusal to use the technology; and the technology being vague, complicated, and not user friendly [7].

Static and interactive health-related mapping reports could generate knowledge, yield proof, and enhance policies [8]. Each interactive mapping report should convey an unambiguous purpose and transmit a flawless meaning to the addressees [9]. Pursuing the health scientists and decision makers, the health-related maps should embrace references of the used data resources and the approach that was used to get the mapped results. The usability of the health-related mapping reports must be accordingly scrutinized and assessed using a representative sample of the potential users before and after releasing the maps [3].

The current scientific literature supports the importance of cooperation between public health scientists and health professionals in integrating health information from diverse sources via portals and applications. These systems can guide public health professionals in designing and developing useful public health policies and interventions [10]. Over the last two decades, the mapping reports have transformed from being static to being dynamic [11]. GIS users prefer interactive reports over static and animated ones [3]. The same literature encourages map developers to consider the practical and social issues of users during development, evaluations, and updates of GIS tools [12].

A number of cancer registries have started interactively mapping their databases’ results, but few of them are assessing the usability and functionality of this technology [13-17]. We are seeking to fill this gap and give an exemplary model to help other registries conduct usability testing studies to tailor their visualized and mapped material according to their potential users’ perceptions and preferences.

This study was the first usability study to assess the quantitative and qualitative metrics data from the sampled health professionals while they are interacting with the published Missouri Cancer Registry and Research Center (MCR-ARC) InstantAtlas mapping reports. Investigators conducted a multiple methodology usability testing study of the published interactive mapping reports of the MCR-ARC. The goals were to understand the ease of use (user friendliness) and user satisfaction with the maps and to measure their effectiveness and efficiency using a convenience sample of health professionals. These maps had been implemented with InstantAtlas (GeoWise Ltd., Scotland); see Multimedia Appendices 1 and 2 [18,19]. The study aims to refine the registry’s published reports to increase the satisfaction of their professional end users. The investigators also wanted to assess whether, and to what extent, the users’ performance would be affected by their demographics, experience, education level, and type of work.

## Methods

### Study Design

The investigators chose a mixed methodology approach. The tested reports had been published on the MCR-ARC website [20]. The researchers conducted a pretest questionnaire, a multi-task usability test, and the System Usability Scale (SUS) for every participant [21].

#### The Pretest Questionnaire

The questionnaire included questions on every participant’s work type, personal information, total experience in the public health field, experience in use of GIS tools, years of practicing public health, and the participant’s education level (see Multimedia Appendix 3). This step was followed by the multi-task test.

#### Multi-Task Usability Test

The multi-task usability test was composed of ten individual tasks that were applied on the tested mapping reports. These tasks were performed by the participants to diagnose the usability of the tested reports. Based on the published mapping reports functionality, the multi-task usability test was constructed by the study investigators to measure the efficiency and effectiveness of the tested reports. The tasks were in the same order for all participants (see Multimedia Appendix 4). The 10 assigned tasks covered most of the maps’ functionality. By conducting all these tasks effectively and efficiently, the users could reach the designer’s expected benefits of our visualized data.

#### The System Usability Scale (SUS)

The System Usability Scale (SUS) is an industrialized and simple 10-item scale to measure the participants’ subjective evaluation of the tested mapping reports’ usability. The SUS was conducted immediately after the completion of the multi-task usability test. The SUS scores range between 0 and 100. Scores above 68 points were counted as acceptable according to usability literature, and higher scores represent the optimal to best score [21].

### Participants

The study’s proposal was approved by the Health Sciences Institutional Review Board (IRB) of the University of Missouri-Columbia. Recruiting emails were sent to faculty in the Master of Public Health (MPH) program and faculty and staff in the School of Medicine’s Department of Health Management and Informatics (HMI) at the University of Missouri-Columbia. Using a convenience sample, investigators ran the study’s trial on the first 7 potential respondents who agreed to participate. The minimum number of participants needed to conduct a successful usability study is 5; a 5-participant study will be able to demonstrate between 55-100% of the usability problems of tested material [22,23]. In this study, we increased the number to 7 subjects to catch more usability issues of our tested reports [24].

### Study Procedure

Every participant tried ten tasks in a safe and private space for an average of 30 minutes per participant. The researchers used a computer laptop to conduct the trial. Windows Media Player software (Microsoft, Washington USA) was installed to record the screen and spoken comments of the participants as they took part in the trial. Task completion time and task completion success were analyzed manually based on the recordings.

The following outcomes were measured:

Performance metrics: A few metrics were utilized to assess the effectiveness and the efficiency of the tested mapping reports and to uncover usability problems. Some of these metrics are defined in terms of critical errors—an error that resulted in an incorrect or incomplete task. If a participant sought help from the test observer to finish a task, it was considered a critical error [25]. The investigators measured the following metrics:

a. Effectiveness: Task completion rate (TCR). TCR is a measure of tasks that were completed without critical errors, and the outputs of the task were correct [24,25]. TCR represented the mapping reports’ usability effectiveness and was analyzed in two distinct ways: by participant and by task.

TCR per participant: The percentage of tasks that were successfully completed by a participant [25].

TCR per task: The percentage of participants who successfully completed a given task [25].

b. Efficiency: The resources expended in relation to the “accuracy and completeness with which users achieve goals” [26]. Using the video records, the time per task was measured from the beginning of the task until the time the participant started the next task.

**Figure 1:**
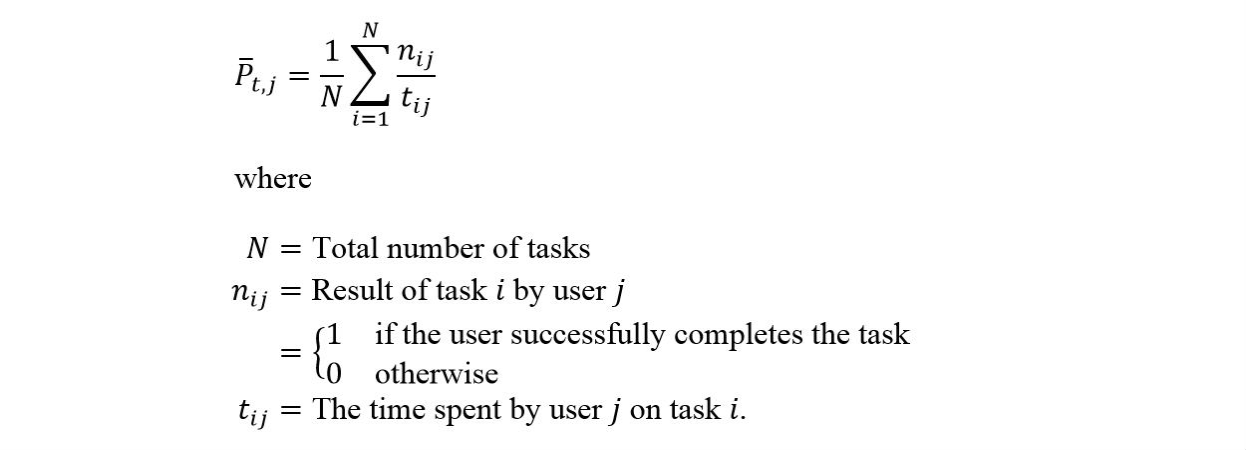
Time-based efficiency (TBE) calculating formula.

The investigators calculated the efficiency and the productivity of the tested mapping reports using the following metrics:

Time-based efficiency (TBE) per task. This is a task-specific version of an overall TBE as shown in Figure 1 [25].

**Figure 2:**
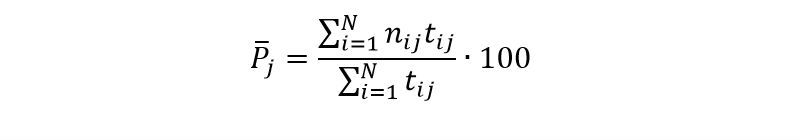
Overall relative efficiency (ORE) per task calculating formula.

Overall relative efficiency (ORE) per task. This is a task-specific version of an overall TBE as defined in [25] and shown in Figure 2.

User satisfaction: Overall satisfaction per study subject was measured by the SUS survey. See the details under study design section.

Before conducting the study, study researchers expected that there would be some factors that might impact the participants’ performance and their satisfaction with the tested maps, and we assessed the influence of these elements on the participants’ performance. These factors were the participants’ education level, work type, experience in health care field, and previous experience with mapping reports and GIS tools [27]. The investigators used a variety of statistical methods, as needed, to explore these relationships (Wilcoxon-Mann-Whitney test, Pearson correlation, and simple linear regression). The Wilcoxon-Mann-Whitney test was conducted using the Web implementation of the method described in the study by Marx et al [28], and the remaining analyses were conducted using Excel (Microsoft). The intended sample size of this study was small since we primarily wished to uncover major usability problems; post-hoc power calculations for simple linear regression with the observed sample data indicate that the power for testing the relationships between the participants’ factors and the TCR or SUS ranged between 3-24% [29]. We used a type I error rate (alpha) of .05 for the hypothesis tests conducted in this project.

## Results

### Participant Demographics

A total of 7 health professionals were interviewed: 1 white male and 6 white females; their ages ranged from the early 30s to late 60s (mean=49.57 years, median=49.17 years). Of the 7 participants, 3 were from the MPH program and 4 were from the HMI department. Four held a doctoral degree in a health care-related field, and 3 had master’s degree in public health, health administration, or health informatics. Furthermore, 5 of the 7 participants were working as research or teaching faculty. Two participants were both staff members and doctoral students in the health informatics program, working in public health research; both had experience in working with mapping reports for at least one year. All 7 participants had experience in the health care field, ranging from 3-38 years (mean=17.8 years, median=13 years). The participants’ total experience in using mapping interactive reports at work ranged between a few months to 15 years (mean=5.6 years, median=2 years). Our participants’ work types can be classified, according to their daily work roles, into two broad categories: Faculty and analysts (n=5) and directors and staff (n=2).

### Reports’ Effectiveness and Efficiency

#### The Mapping Reports’ Effectiveness

##### Effectiveness per Participant

A PhD-holding participant, who had 13 years of experience in the public health field and in GIS use, could not accomplish two of the assigned tasks because she “had no idea how to navigate them” as she commented. Three of the remaining 6 participants—a PhD-holding faculty member and two staff members—were not able to follow expected pathways to finish the assigned tasks and got false results for some tasks; these participants thought that they completed the tasks successfully and did not ask for help or clarification. All 3 participants had 1-6 years’ experience using GIS tools. Of the remaining 3 participants, all completed the tasks effectively and efficiently, including one who had the least amount of experience with mapping reports and tools of the 7 participants.

**Figure 3:**
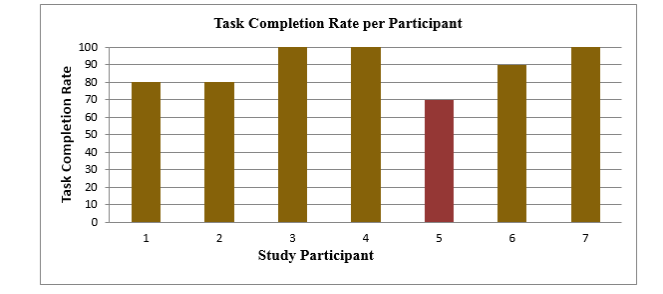
Task completion rate (TCR) per participant. Dark gold indicates participants who finished the trial with >78% TCR; red indicates participants who finished the trial with <78% TCR.

The effectiveness was defined as: “The accuracy and completeness with which users achieve specified goals” [26]. The results in our study ranged from 70-100%, with only 1 participant finishing the trial with a TCR <78% (Figure 3), 78% is the minimum TCR score accepted by some scholars [25]. Four of the 7 subjects attained a TCR of 90% or more.

##### Effectiveness per Task

**Figure 4:**
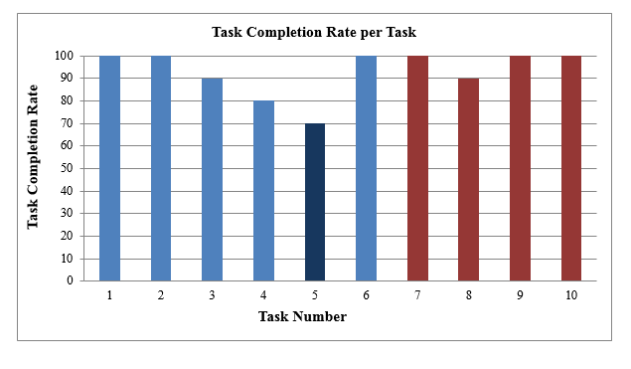
Task completion rate (TCR) per task. Blue indicates tasks involving the area health profile (Tasks 1-6); red indicates tasks involving the double map (Tasks 7-10). Light blue indicates tasks that had >78% TCR; dark blue indicates the task (Task 5) with <78% TCR.

The investigators used the task completion formula to measure the TCR by task. The results are shown in Figure 4. Six of the ten assigned tasks reached a TCR of 100%, and two of the ten tasks had a TCR of 90%, whereas one task had a TCR of 80%, and the remaining task had a TCR of 70%.

Task numbers 1, 2, 7, 9, and 10 were very simple, such as open or close a functional button on the reporting map. All had a TCR of 100%.

Task numbers 3, 4, 5, and 8 got lower TCRs than the previously mentioned tasks, with scores ranging between 70-90%. Before conducting this study, the study investigators ranked task numbers 3, 4, 5, and 8 along with task number 6 as complicated tasks that need specific skills and knowledge to be completed successfully. One complicated task, number 6, was completed effectively by all subjects.

#### Mapping Reports’ Efficiency and Productivity

The time per tasks ranges from the minimum 2 seconds for task number 10 to the maximum 297 seconds for task number 8, which included three subtasks. As seen in Table 1, even among the tasks with a 100% completion rate, there was variation in the time spent by the participants. The median time on task number 6 was the highest, followed by task numbers 8, 5, 4, and 3, respectively; this was relatively related to the complexity of the tasks.

**Table 1:** Time on study tasks.

Task #	Task time range (seconds)	Task time mean (seconds)	Task time median (seconds)
1	3-62	19.4	12
2	10-51	23	16
3	13-64	34.8	33
4	20-87	57	53
5	11-268	95.8	75
6	46-215	136	145
7	2-20	11	11
8	90-297	165.1	141
9	3-16	41.5	21
10	2-38	3.2	3

##### Time-Based Efficiency (TBE) and Overall Relative Efficiency (ORE)

**Figure 5:**
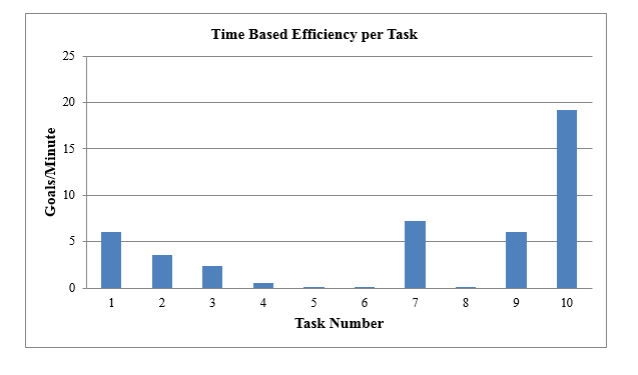
Time-based efficiency (TBE) per task.

**Figure 6:**
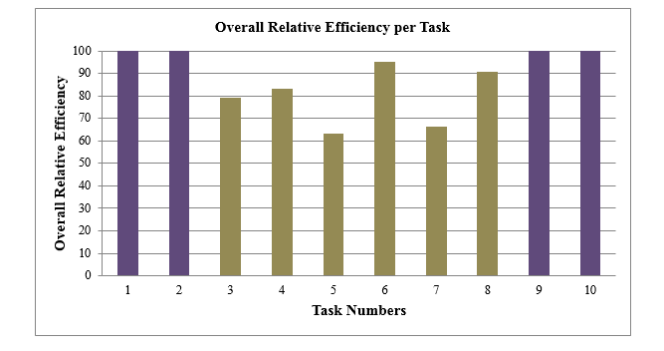
Overall relative efficiency (ORE) per task. Purple indicates tasks with 100% ORE per task, dark tan indicates tasks with less than 100% ORE per task.

Figures 5 and 6 show the time-based effeciency (TBE) and the overall relative effeciency (ORE) for each of the tasks.

From Figure 5, we can see that the TBE per task varied for the ten tasks. Task number 10 had the highest TBE (19.2 goals/min); this result conforms to the simplicity of the task (close the map). It is followed by task numbers 7, 9, and 1, which are also simple tasks (proceed to the “double map” link on the desktop, open the “area profile,” map link in the desktop, and check the sources of our mapping report data, respectively). Task numbers 2, 3, and 4, all complicated tasks, had very low TBE rates. Task numbers 5, 6, and 8 were the most complicated tasks; they had the lowest TBE levels.

Figure 6 shows that the highest ORE rates were for task numbers 1, 2, 9, and 10; they were all simple tasks. Task number 6 had about 97% ORE rate despite it being ranked as one of the complicated tasks. Task numbers 3, 4, 5, and 8 had the lowest ORE per task.

### Users’ Satisfaction

**Figure 7:**
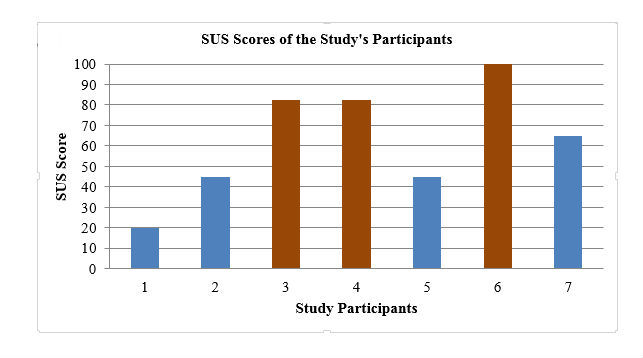
System Usability Scale (SUS) scores of the study’s participants. Brown color indicates SUS score of >68 points, and blue color indicates SUS score of <68 points.

SUS is a standard 10-question questionnaire given to every participant after the tasks to measure user satisfaction with the tested maps [20]. As Figure 7 shows, the SUS score range for all the participants was 20-100 points with an average of 62.86 points and a median of 50.50 points. The SUS scores for 3 of the 7 study participants were above the target of 68 points, and they were satisfied with the maps they tested. The remaining 4 of our participants’ scores were below 68 points. The interpretation of the SUS scores for the study subjects ranged between worst imaginable to best imaginable, and according to the school grade scale, the scores were between A and F with an average of D.

### Factors Affecting the Participants’ Performance

As discussed in the methods section, we expected that there are some factors that could impact the participants’ performance and their satisfaction with the tested interactive mapping reports.

#### Education Level and Work Type Factors

We assessed whether the education level of the participants impacted the distribution of either their TCR or SUS score using the Wilcoxon-Mann-Whitney test [27]. We classified the participants as PhD or master degree holder subjects, and we tested these two groups’ TCR. We did not find any statistically significant difference in the distribution of the TCR by education level (*P*=.91). Also, there was no statistically significant difference in the distribution of the SUS score by education level (*P*=.82).

The Wilcoxon-Mann-Whitney test was used also to assess whether the participants’ distribution of TCR differs by their work type. We categorized the participants into two groups: a faculty and analysists group and a staff and directors group. The difference in the distribution of TCR between the two groups was statistically insignificant (*P*=.75).

**Table 2:** Demographic and previous expertise factors of the study participants versus the trial’s task completion rate (TCR) and the participants’ System Usability Scale (SUS) scores.

The studied factors	*P* value
Education level versus TCR^a^	.91
Education level versus SUS^b^ score	.82
Work type versus TCR	.75
Previous experience in health care field versus TCR	.70
Previous experience in GIS^c^ use versus TCR	.03
Previous experience in health care field versus SUS score	.82
Previous experience in GIS use versus SUS score	.17

^a^TCR: task completion rate.

^b^SUS: system usability scale.

^c^GIS: geographic information system.

**Table 3:** Correlation between the studied usability elements (effectiveness, efficiency, and satisfaction).

The studied factors	Correlation coefficient	*P* value
TCR^a^ per participant versus SUS^b^ score	.70	.08
TCR per task versus TBE^c^	.50	.25
TCR per task versus ORE^d^	.92	*P*>.0.01
Efficiency per participant^e^ versus SUS score	.70	.07

^a^TCR: task completion rate.

^b^SUS: System Usability Scale.

^c^TBE: tine-based efficiency.

^d^ORE: overall relative efficiency.

^e^The total time in seconds of the whole trial per participant.

#### Experience in the Health Care Field and Experience With Mapping Reports and Geographic Information Systems (GIS) Tools

We conducted simple linear regressions to explore the relationship between the TCR of the study subjects on the usability test and between both experience in the health care field and previous experience with mapping reports and other GIS tools. The relationship between the TCR and experience in the health care field was insignificant (*P*=.70). There was a statistically significant relationship between the subjects’ TCRs and experience using GIS tools (*P*=.03). There was no statistically significant relationship between the SUS levels and previous experience in the health care field or with GIS tools for the study participants. The *P* values for these results were (*P*=.82) and (*P*=.17), respectively.

Table 2 has the results from studying the demographics and experience in the health care field and experience with GIS tools versus their TCR and SUS scores of the trials they performed in this study.

### Correlation Between the Studied Usability Elements (Effectiveness, Efficiency, and Satisfaction)

As Table 3 shows, we studied the relationship between the TCRs and the SUS scores, and this revealed a positive, but statistically insignificant, correlation between the two studied factors (*r*=.70, *P*=.08). The relation between the TCR and both the TBE and the ORE factors were explored. The results revealed that there were positive correlations between the effectiveness (TCR) and both the efficiency in terms of TBE (albeit statistically insignificantly) and ORE (statistically significant) for the studied maps (*r*=.50, *P*=.25 and *r*=.92, *P* P>.0.01, respectively). There was a positive, but statistically insignificant, correlation between the time spent by the participants for all tasks and the SUS scores they gave after they finished the test. The correlation was positively strong (*r*=.70, *P*=.07).

## Discussion

### Main Findings

This study concluded that the tested maps should undergo extensive refining using a user-centered approach to overcome the discovered usability issues. This approach could enable map designers to facilitate good user-software interaction and usability. This will let the designers meet their maps’ potential users’ expectations [30]. Usability testing studies should be conducted before and after releasing the maps to their potential users.

### Effectiveness and Efficiency

#### Effectiveness per Participant

In any usability study, the investigators should always aim for a 100% TCR per participant; however, some usability scholars consider a TCR of ≥78% per participant acceptable [25]. Six of the 7 participants exceeded the target TCR per participant of 78%, and just 1 participant out of the 7 got a rate less than 78% (Figure 3). These results reveal that the trial was carried out effectively by 6 of the total 7 participants. Surprisingly, a PhD-holder participant with years of experience in the public health field and in GIS use could not accomplish two of the assigned tasks, whereas other participants with lower education and null experience handled the trial effectively.

Three participants incorrectly thought that they had effectively completed some tasks because there were no alerts or pop-ups to make them aware that they made mistakes. Some tasks were not dependent on each other, so the participants were not interrupted if the task was wrongly handled. Also, some of these tasks need to be answered by writing on paper and needed specific cognition and knowledge to be answered.

#### Effectiveness per Task

Our results support the scientific evidence from a study conducted in 2006 that concluded that technology effectiveness is affected by task complexity factor [31]. Task numbers 1, 2, 7, 9, and 10 were very simple, such as open or close a functional button on the reporting map. These tasks did not require that participants find or interpret complicated epidemiologic or statistical results so all the participants were able to complete these tasks successfully.

All the subjects accomplished task number 6 effectively, although it is categorized among the trial’s complicated tasks. This could be due to the study subjects’ previous experience in public health and health care; also, all the subjects were epidemiologists or researchers familiar with biostatistics and epidemiology. Additionally, it may be because the task is very connected to the preceding tasks and it was very easy to accomplish when they solved the previous tasks.

Not surprisingly, the remaining complicated tasks, numbers 3, 4, 5, and 8, received the lowest TCR scores. Participants who lacked specific skills and knowledge were unable to complete these tasks successfully.

According to the study subjects’ comments and by reviewing the recorded trial videos, additional usability issues with the published maps were revealed. These usability problems explained why these tasks were hard to be accomplished even with expert participants in public health, in the health care field, and in GIS tool use. The maps’ designer has refined the maps according to comments made by the participants and rereleased them.

#### Efficiency

From Table 1, we determined that even for the tasks that were ranked easy and uncomplicated, some study subjects took more time and effort to get the tasks successfully conducted than others. This might need usability adjustment by the tested maps’ designer in the future so these tasks could be completed by all users within comparable times.

From the TBE results (Figure 5), we expected that in addition to the cognition and knowledge needed to accomplish these tasks, usability issues we discovered in this study might make these tasks even more complicated than the investigators thought. The ORE results supported previous literature’s findings that the efficiency is relatively associated with the complexity of these tasks [31].

After reviewing the recorded videos, the primary investigator concluded that task number 6 was easy to handle by the study subjects because it was closely related to its preceding three tasks. The study’s audio-video recordings revealed that repeating and retrying the foregoing tasks allowed task number 6 to be accomplishment by all the participants.

### Participants’ Satisfaction

Based on the SUS scores, we demonstrated that we need to consider participants’ comments and refine our tested maps in order to make potential users more satisfied and pleased.

We were surprised that many of the SUS scores were very low; this reinforces the need to test systems on potential users rather than assuming they will find the system usable. To improve user satisfaction, we are willing to consider all the participants’ comments to refine the tested maps. User surveys already were available on the reports to assess the users’ satisfaction and collect their feedback on using the mapping reports. The study investigators have made modifications according to this study’s participants’ comments, but this remodeling has not yet resulted in much feedback. The mentioned modifications could improve the evaluated reports and might make published reports more understandable and usable and could increase the users’ satisfaction [32].

### Factors Affecting the Participants’ Performance

As the study researchers expected, there was a statistically significant relationship between the subjects’ TCRs and their experience in using GIS tools. So, there is dependency of the TCRs on the participants’ previous experience with GIS technology. This finding supports the findings of 2 previous studies revealing that the performance of users on a specific technology are related to previous exposure to that technology [27,33]. Also, these results supported previous findings of several studies concluding that experience and knowledge affects the task success rates of the tested technology [34,35].

The investigators did not find any statistically significant relationship between education level in terms of the graduate degree the participant holds and participants’ TCRs. There was no statistically significant relationship between participants’ education level and their SUS scores. The relationship between participants’ TCRs on the test and their work type was statistically insignificant. The relation between participants’ TCRs and between their experiences in the health care field was statistically insignificant. The study failed to discover any statistically significant relationship between SUS levels and both TCRs and previous experience with GIS tools for the study participants.

### Correlation Between the Studied Usability Elements (Effectiveness, Efficiency, and Satisfaction)

The results revealed strong correlation between the three usability elements. The results support our assumption that the user will be satisfied if they can conduct the trial effectively and efficiently.

### Strengths and Limitations of the Study

This is the first usability study to assess published MCR-ARC InstantAtlas reports. This is a good first step; these results might be generalized to assess the usability of all MCR-ARC’s mapping reports as well as GIS reports published elsewhere.

The 7 participants were all health professionals from academic departments. The small sample size coupled with the use of a nonprobability convenience sample of academic health professionals limits the generalizability of these results.

The video records were reviewed manually by one of the investigators. This study could not capture all the performance and behavior of the participants while they were interacting with the tested maps. A better way to capture participants’ awareness and cognitive processes would be to make use of an eye tracking system. The investigators are thinking of using advanced usability software to track user behavior in the future.

### Ongoing Work and Recommendations

The investigators conducted a second round of the usability study using professionals who are working directly in day-to-day cancer research and policy after a revision to published maps due to this first round. The researchers assumed that second round professionals might have more valuable perspectives and insights toward the tested GIS reports. The investigators conducted the second-round study after considering the first round participants’ responses and suggestions. All the future MCR-ARC mapping reports’ usability should be assessed during the designing process and after publishing the maps. The investigators should use advanced usability tools to test the published maps.

### Conclusions

The three main elements of the tested mapping reports’ usability were measured and assessed by this study in terms of effectiveness, efficiency, and user satisfaction. The tested maps’ effectiveness outcomes were better than the efficiency and satisfaction outcomes. The trial was conducted effectively by 6 of the total 7 participants. The study discovered that effectiveness and efficiency metrics were related to the given tasks’ complexity; easier tasks were accomplished more effectively and efficiently than complicated tasks. Although most of the study subjects accomplished most of the tasks effectively and efficiently, the users’ satisfaction was surprisingly poor.

This study revealed that there was a statistically significant relationship between the subjects’ performance on the study test and their experience using GIS tools.

The study researchers discovered that the pretest questionnaire and the multi-task usability test were not enough to discover all the usability issues of the tested maps. Seeking users’ text comments and analyzing the video recordings are very valuable in exploring more usability concerns and in revealing potential users’ preferences and perspectives toward GIS tools and maps. The study revealed that to facilitate good map-user interaction and usability, designers need to conduct usability trials on the maps, including the maps’ potential users before and after publishing them.

This study’s results might be generalized to other mapping reports and might be used to refine the usability and functionality of these reports as well as other GIS reports and tools of the MCR-ARC. The study findings might point the importance of including GIS tools’ end users in the basic stages of designing and developing GIS tools.

## Appendix

Multimedia Appendix 1 Cancer, demographics, and behavioral risks—area health profile report.

Multimedia Appendix 2 Cancer, demographics, and behavioral risks—double map report.

Multimedia Appendix 3 Pretrial questionnaire.

Multimedia Appendix 4 Multi-task usability testing.

## Abbreviations

GIS: geographic information system
HMI: Health Management and Informatics
IRB: institutional review board
MCR-ARC: Missouri Cancer Registry and Research Center
MPH: Master of Public Health
ORE: overall relative efficiency
SUS: System Usability Scale
TBE: time-based efficiency
TCR: task completion rate
