# Supplementary figures and images for: Usability Assessment of the Missouri Cancer Registry’s Published Interactive Mapping Reports: Round One
Source: JMIR Hum Factors. 2017 Aug 4;4(3):e19. doi: 10.2196/humanfactors.7899 (PMC5562933; doi:10.2196/humanfactors.7899)

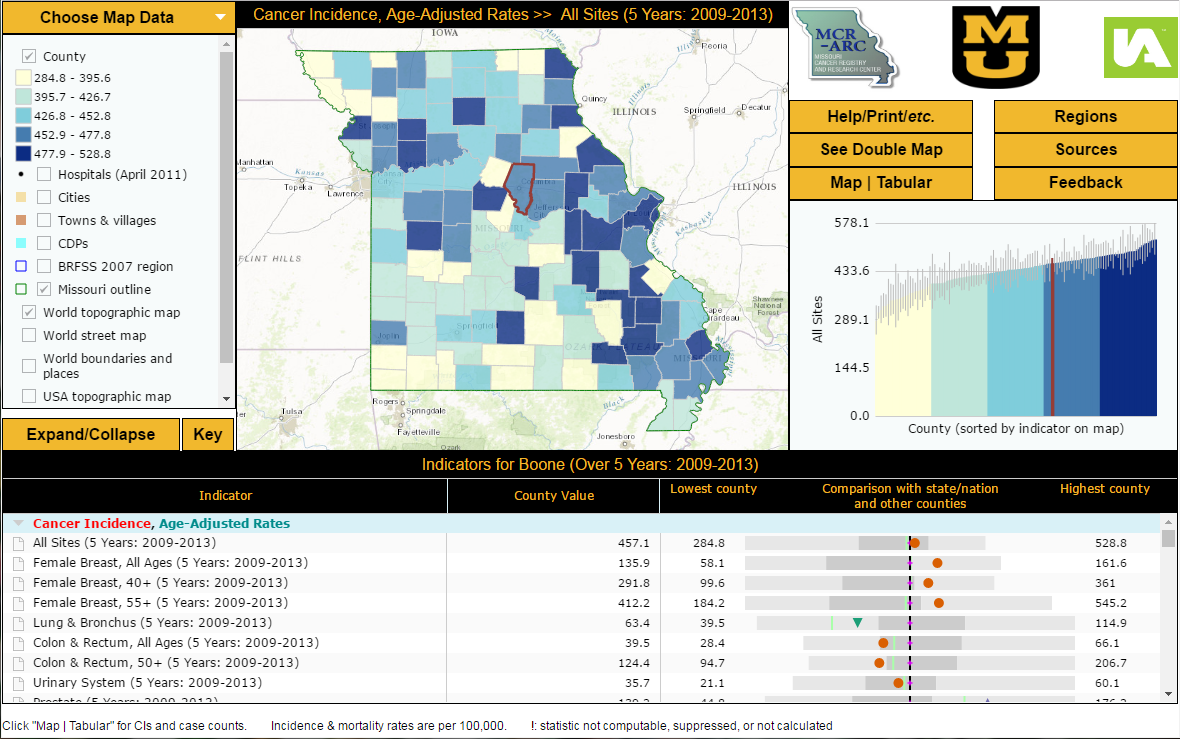

Supplement: Multimedia Appendix 1 [file humanfactors_v4i3e19_app1.PNG]

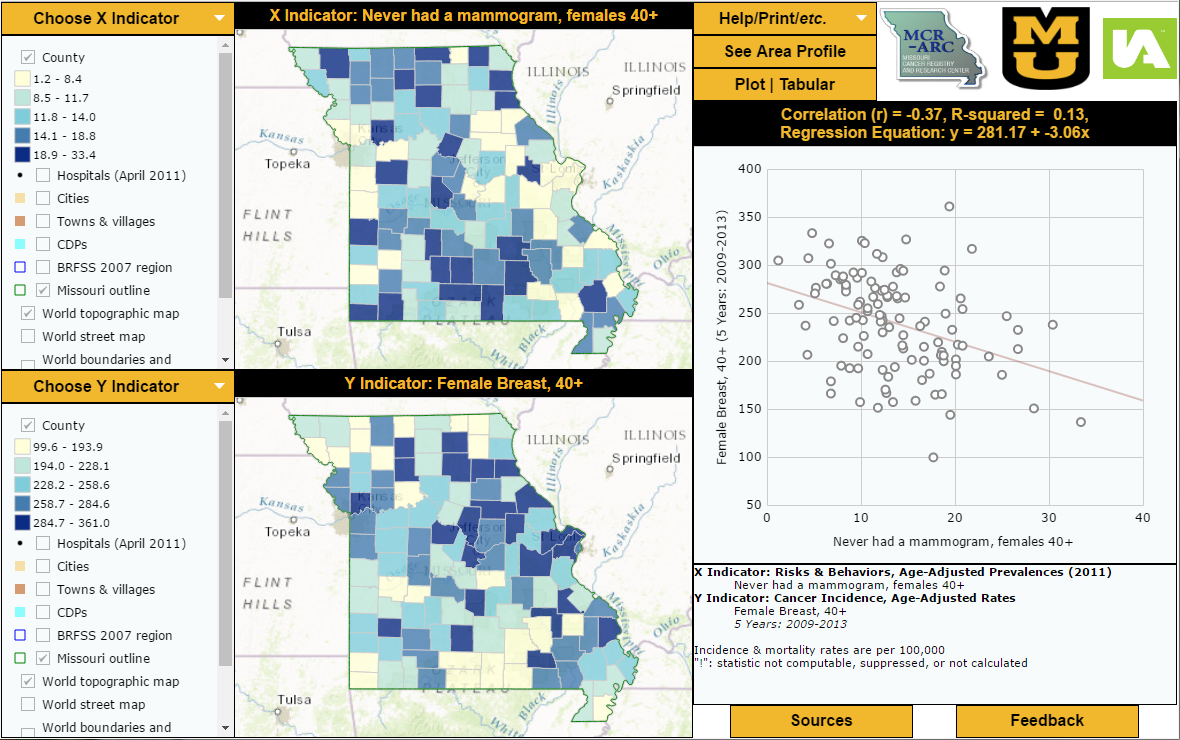

Supplement: Multimedia Appendix 2 [file humanfactors_v4i3e19_app2.PNG]

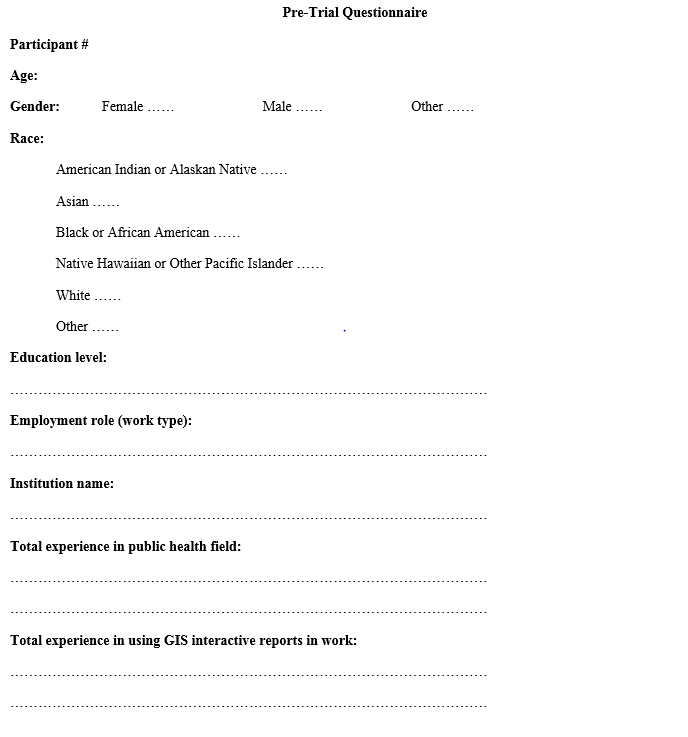

Supplement: Multimedia Appendix 3 [file humanfactors_v4i3e19_app3.PNG]

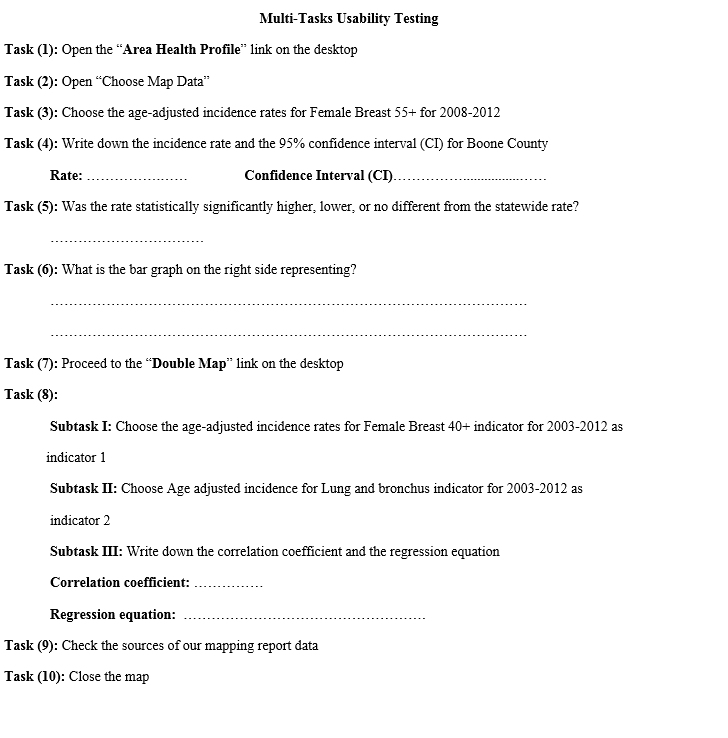

Supplement: Multimedia Appendix 4 [file humanfactors_v4i3e19_app4.PNG]
